# Supplementary material for: Validation of the Health Index in the Postoperative Period: Use of the Nursing Outcome Classification to Determine the Health Level
Source: Healthcare (Basel). 2024 Apr 20;12(8):862. doi: 10.3390/healthcare12080862 (PMC11050318; doi:10.3390/healthcare12080862)
Supplement: Supplementary file 1 [file healthcare-12-00862-s001.zip › Supplementary Tables.pdf]

Supplementary Table S1. Health Variables Definition [29]

Health Variables Definition
Physical Functioning (Pf): Functioning of the apparatuses and corporative systems of the person: locomotor, digestive, respiratory, urinary, genital, endocrine and circulatory systems and bone, muscular and nervous systems. It has a reversible character.
Mental Functioning (Mf): In order to determine the mental functioning, the state of consciousness, space-time orientation, behavior and language will be valued. It is reversible.
Social Functioning (Sf): To determine the social functioning of a person will be assessed the ability to communicate and interact with other people. It is reversible.
Comfort Status (Cs): The welfare of the person, their tranquility, personal security, adaptation to the environment will be valued.
Material Resources (Mr): Set of material goods needed to live.
Time Resource (Tr): Temporary availability to carry out your care.
Sings Presence (Sg): Sign measurable and valuable by the healthcare professional (heart rate, blood pressure ...)
Symptoms Presence (Sn): Manifestations of each person (pain, nausea ...)
Physical Condition (Pc): Sequel of irreversible character in the functioning of the body.
Mental Condition (Mc): Sequel of irreversible character in mental functioning.
Social Condition (Sc): Sequel of irreversible character in social functioning.

Supplementary Table S2. Validation of the correlation between Health Variables and NOC labels

Health Variables	NOC selected in Construction of a preliminary instrument phase	Internal Validation Round 1	Internal Validation Round 2
		Interpretation	Final decision
**Physical Functioning**	Ambulate	Not approved	
	Self-care: Activities of daily living	Not approved	
	Self-care: Instrumental activities of daily living	Not approved	
	Personal welfare	Revised	Not approved
	Health promoting behavior	Not approved	
	Bowel continence	Not approved	
	Urinary continence	Not approved	
	Physical aging	Not approved	
	Comfort status	Revised	Not approved
	Physical comfort state	Not approved	
	Personal health status	Approved	Approved
	Physical comfort state	Not approved	
	Skeletal function	Not approved	
	Gastrointestinal function	Not approved	
	Renal function	Not approved	
	Auditory sensory function	Not approved	
	Skin sensory function	Not approved	
	Sensory function taste and smell	Not approved	
	Sensory proprioceptive function	Not approved	
	Sensory function vision	Not approved	
	Sensory function	Not approved	
	Mobility	Not approved	

	Discomfort level	Not approved	
	Health orientation	Revised	Not approved
Mental Functioning	Self-care: Activities of daily living	Not approved	
	Self-care: Instrumental activities of daily living	Not approved	
	Self esteem	Not approved	
	Cognition	Not approved	
	Comfort status	Revised	Not approved
	Psychospiritual comfort state	Not approved	
	Personal health status	Approved	Approved
	Neurological status	Not approved	
	Discomfort level	Not approved	
	Health orientation	Revised	Not approved
	Health promoting behavior	Not approved	
Social Functioning	Acceptance: State of Health	Not approved	
	Quality of life	Revised	Not approved
	Health promoting behavior	Not approved	
	Comfort status	Revised	Not approved
	Sociocultural comfort state	Not approved	
	Personal health status	Approved	Approved
	Health orientation	Revised	Not approved
	Severity of loneliness	Not approved	
	Social support	Not approved	
Comfort Status	Acceptance: State of Health	Not approved	
	Adaptation to physical disability	Revised	Not approved
	Self esteem	Not approved	
	Personal welfare	Revised	Not approved
	Quality of life	Revised	Not approved
	Health promoting behavior	Not approved	
	Comfort status	Approved	Approved
	Health orientation	Revised	Not approved
Material Resources	Self-care: Instrumental activities of daily living	Not approved	
	Personal welfare	Not approved	
	Quality of life	Revised	Not approved
	Health promoting behavior	Not approved	
	Health beliefs: resource perception	Approved	Approved
	Health orientation	Revised	Not approved
Time Resource	Self-care: Instrumental activities of daily living	Revised	Not approved
	Personal welfare	Not approved	
	Quality of life	Revised	Not approved
	Health promoting behavior	Not approved	
	Health beliefs: resource perception	Approved	Approved
	Health orientation	Revised	Not approved
Sings Presence	Knowledge: disease process	Approved	Approved
	Patient / user satisfaction: Symptom control	Not approved	
	Symptom severity	Revised	Not approved
	Knowledge: disease process	Approved	Approved

**Symptoms Presence**	Symptom control	Revised	Not approved
	Patient / user satisfaction: Symptom control	Not approved	
	Symptom severity	Revised	Not approved
**Physical Condition**	Adaptation to physical disability	Revised	Not approved
	Ambulate	Not approved	
	Comfort status	Revised	Not approved
	Personal health status	Approved	Approved
	Physical form	Not approved	
	Skeletal function	Not approved	
	Mobility	Not approved	
**Mental Condition**	Self esteem	Not approved	
	Comfort status	Revised	Not approved
	Personal health status	Approved	Approved
	Neurological status	Not approved	
	Cognition	Not approved	
**Social Condition**	Acceptance: State of Health	Not approved	
	Quality of life	Revised	Not approved
	Comfort status	Revised	Not approved
	Personal health status	Approved	Approved
	Social support	Not approved	
